# Professional killers: The role of extracellular vesicles in the reciprocal interactions between natural killer, CD8+ cytotoxic T‐cells and tumour cells

**DOI:** 10.1002/jev2.12075

**Published:** 2021-04-01

**Authors:** Filippo Del Vecchio, Verena Martinez‐Rodriguez, Monique Schukking, Alexander Cocks, Elisabetta Broseghini, Muller Fabbri

**Affiliations:** ^1^ University of Hawai'i Cancer Center Cancer Biology Program University of Hawai'i at Manoa Honolulu Hawaii USA; ^2^ Department of Cell and Molecular Biology John A. Burns School of Medicine University of Hawai'i at Manoa Honolulu Hawaii USA; ^3^ Department of Molecular Biosciences & Bioengineering University of Hawai'i at Manoa Honolulu Hawaii USA; ^4^ Department of Experimental, Diagnostic and Specialty Medicine (DIMES) University of Bologna Bologna Italy

**Keywords:** cancer, CTLs, extracellular vesicles, immune system, NK cells

## Abstract

Extracellular vesicles (EVs) mediate the cross‐talk between cancer cells and the cells of the surrounding Tumour Microenvironment (TME). Professional killer cells include Natural Killer (NK) cells and CD8+ Cytotoxic T‐lymphocytes (CTLs), which represent some of the most effective immune defense mechanisms against cancer cells. Recent evidence supports the role of EVs released by NK cells and CTLs in killing cancer cells, paving the road to a possible therapeutic role for such EVs. This review article provides the state‐of‐the‐art knowledge on the role of NK‐ and CTL‐derived EVs as anticancer agents, focusing on the different functions of different sub‐types of EVs. We also reviewed the current knowledge on the effects of cancer‐derived EVs on NK cells and CTLs, identifying areas for future investigation in the emerging new field of EV‐mediated immunotherapy of cancer.

## 1 INTRODUCTION

The immune system is made up of specialized cells that protect body homeostasis when infectious or tumour threats try to destroy its integrity. Among the plethora of cells alerted to intervene, natural killer (NK) and cytotoxic CD8+ T (CTLs) cells are deemed as the ‘professional killers’ because of their involvement in the direct killing of pathogens. These lymphocytic cells share common progenitors and immune functions (Trinchieri, 1989). NK cells and CD8+ T cells engage directly pathogenic cells and cause their death releasing cytotoxic molecules but they can also produce regulatory factors to stimulate other immune players, thus participating in the concerted effort of pathogen destruction. However, their activation mechanisms substantially differ: while CD8+ T cells need to be primed and activated by other immune cells, the so‐called antigen‐presenting cells (APC), which present pathogenic antigens to them, NK cells are able to kill without prior antigen recognition, thus their attribute of ‘natural killers’ (Trinchieri, 1989; Zhang & Bevan, 2011). More recently, this stark distinction started to blur and now it is believed that also NK cells might possess some features traditionally showed by B and T cells such as memory and adaptation (O'Sullivan et al., 2015; Vivier et al., 2011). Extracellular vesicles (EVs) are membrane‐enclosed particles released by almost every cell type in the extracellular space during physiological and pathological conditions. Their heterogeneity in terms of size and shape led to the definition of some classification criteria that essentially distinguish these vesicles based on their size and biogenesis mode. Despite the efforts from the International Society for Extracellular Vesicles (ISEV) to standardize their definitions, some degree of freedom still exists when it comes to present research data related to EVs generating confusion and uncertainty (Lötvall et al., 2014; Théry et al., 2018). The great significance attributed to EVs is related to the presence of bioactive molecules such as nucleic acids and proteins carried as cargo inside or on the surface of the EV and the possibility of their transmission from one cell to another, thereby impacting the functions of the recipient cells. Indeed, EVs have been described as a new mode of communication between cells both locally and distally, a discovery that fuelled research on the subject (Valadi et al., 2007). A large portion of this research has been devoted to the study of EVs roles in cancer and the effects of EV exchange between cancer and immune cells. With abundant attention on the influence exerted by tumour EVs on immune cells, limited number of information is presently available on the role of immune cell‐derived EVs and their effects on tumour cells. In this review, we propose to bridge this gap exposing the results of recent studies aimed at investigating the impact produced by EVs from NK and CTLs on cancer cells. At the same time, by giving account of the latest findings on tumour‐derived EVs and their effects on the aforementioned immune cells, we wish to provide a complete view of the roles of these vesicles in the continuous battle between immune professional killers and cancer cells.

## 2 NATURAL KILLER CELLS

Natural Killer (NK) cells have been first identified in the 1970s as naturally cytotoxic killer lymphocytes that can kill the target cell without prior exposure to antigens (Kiessling et al., 1975). They make up for about 10% of all peripheral blood lymphocytes and are characterized by the expression of CD56 and lack of CD3 cell surface proteins (Robertson & Ritz, 1990).Given their ability to secrete interferon‐γ (IFN‐γ), NK cells are defined as prototypic cytotoxic members of Group 1 innate lymphocyte cells (ILCs) (Spits et al., 2013).

### 2.1 Generation and maturation

NK cells develop in the bone marrow as well as in the secondary lymphoid tissues (SLTs) including tonsils, spleen and lymph nodes (LNs) (Scoville et al., 2017; Seaman et al., 1978). Regardless of the location of origin, maturation of NK cells from hematopoietic stem cells (HSCs) has been divided into 6 coordinated stages, all of which are characterized by the loss or expression of distinct cell surface proteins (Scoville et al., 2017; Yu et al., 2013). The process results in the generation of two populations of mature NK cells: CD56^bright^ and CD56^dim^. Most NK cells in the circulation (∼90%) are CD56^dim^, whereas CD56^bright^ NK cells (∼5%) reside primarily in the SLTs (Poli et al., 2009). CD56^bright^ NK cells are primary producers of inflammatory cytokines, including IFN‐γ, TNF‐ß, IL‐10, IL‐13, and GM‐CSF, while the CD56^dim^ subset of NK cells exhibits a stronger cytolytic function (Cooper et al., 2001; Jacobs et al., 2001). Maturation starts when Lin^−^CD34^+^ HSCs differentiate into a CD45RA^+^ lymphoid‐primed multipotential progenitor (LMPP) (Abel et al., 2018). Expression of CD7, CD10, CD38 and CD127 (IL‐7Rα) further converts LMPPs into a common lymphoid progenitor (CLP) that can still differentiate into other cell types, including B‐cells, T‐cells, NK cells and other ILCs. Downregulation of CD127 and upregulation of CD122 converts CLPs into NK cell‐restricted progenitor (NKP) (Renoux et al., 2015). The most important changes in the maturation process of NKP cells include the gradual downregulation of CD34 and CD117, followed by the upregulation of CD94, responsible for the differentiation into the CD56^bright^ subpopulation. Then, CD56^bright^ NK cells further differentiate into CD56^dim^ NK cells by upregulating CD16 and killer cell Ig‐like receptors (KIRs) (Freud & Caligiuri, 2006; Freud et al., 2006). NK cell development is driven by a variety of signals generated by cytokines and transcription factors. For example, transition of HSCs into CLPs is promoted by IL‐3, whereas IL‐15 is required for the differentiation of CLPs into mature NK cells (Imada et al., 1998; Kovanen & Leonard, 2004; Muench et al., 2000; Reddy et al., 2000). Another example includes the transcription factor ETS1 that has been found to be an important regulator of human NK cell development and differentiation by directly stimulating the expression of other key transcription factors that are indispensable for NK cell development including E4BP4, TXNIP, TBET, GATA3, HOBIT and BLIMP1 (Taveirne et al., 2020).

### 2.2 Activation and killing mechanisms

An NK cell killing mechanism was first proposed in the early 1980s as the missing self‐hypothesis that states that NK cells are able to recognize and kill target cells lacking expression of self major histocompatibility complex (MHC) class I molecules (Kärre et al., 1986; Ljunggren & Kärre, 1990). It is now clear that a variety of activating and inhibitory receptors interacting with MHC class I molecules, MHC class I–like molecules, and other MHC‐unrelated proteins on target cells could also regulate NK cell activation (Caligiuri, 2008). In physiological conditions, inhibitory receptors such as the inhibitory killer immunoglobulin‐like receptors (KIRs) binding to classical MHC class I ligands (HLA‐A,‐B,‐C), and the heterodimeric receptors CD94‐NKG2A binding to non‐classical MHC class I ligands (HLA‐E) send inhibitory signals to the NK cell upon interaction with MHC class I‐expressing cell in order to prevent killing of the target cell (Aribi, 2017; Bryceson et al., 2006; Moretta et al., 1996). However, cells with lower or no expression of MHC class I proteins, such as tumour cells or virus‐infected cells, cause a decrease of these inhibitory signals (Hicklin et al., 1999; Koutsakos et al., 2019). On the other hand, the cellular stress caused by these cells leads to an upregulation of activating ligands on their cell surface which in turn interact with activating receptors on NK cells and ultimately cause the killing of the target cells via NK cell‐mediated cytotoxicity or, indirectly, via the secretion of pro‐inflammatory cytokines (Chan et al., 2014). This case is exemplified by the NKG2D homodimers that bind to the stress‐induced molecules MICA and MICB (MHC class I chain‐related proteins A and B) as well as ULBPs (UL16‐binding proteins) (Cosman et al., 2001; Groh, 1998). Another group of activating receptors are the natural cytotoxicity receptors (NCR) including NKp46, NKp30, and NKp44 that belong to the immunoglobulin superfamily (Kruse et al., 2014). Additionally, the interplay between activating receptors and pro‐inflammatory cytokines such as IL‐12, IL‐15, IL‐18, IL‐21 and IFN‐γ constitutes another modality of NK cells activation (Biron et al., 1999). NK cells use three fundamental mechanisms to kill their target cells. They store cytotoxic molecules including granzymes and perforin in secretory lysosomes (Lettau et al., 2007). Upon recognition of a target cell and subsequent activation, the NK cell induces exocytosis of the secretory lysosomes releasing their content into the target cell. Perforin enables entry of granzymes into the target cell where it activates pathways of cell death that subsequently leads to the killing of the target cell (Chowdhury & Lieberman, 2008; Pipkin & Lieberman, 2007). The second killing mechanism is mediated through the expression of FAS ligand (FASL) or tumour‐necrosis‐factor‐related apoptosis‐inducing ligand (TRAIL), a molecule belonging to the TNF‐proteins superfamily. These ligands bind to receptors on the surface of virus‐infected cells or tumour cells causing cell death of the target cells by activation of the apoptotic caspase machinery (Ashkenazi, 2002). Lastly, secretion of cytokines by the NK cell has been found to indirectly kill target cells by stimulating other cells of the immune system. For example, NK cells‐secreted IFN‐γ promotes the differentiation of CD4^+^ T cells into T helper cells (Th1) which in turn promote CTL differentiation (Scharton & Scott, 1993). Furthermore, NK cells are able to prime dendritic cells to induce protective CD8^+^ T cell responses via IFN‐γ production, or by activation of antigen‐presenting cells in order to up‐regulate MHC class I expression and thereby increase the antiviral response (Mocikat et al., 2003; Wallach et al., 1982).

## 3 CYTOTOXIC CD8+ T CELLS

### 3.1 Generation and activation

Cytotoxic CD8+ T lymphocytes (CTLs) are part of the adaptive immune system. Their generation process begins from the hematopoietic stem cells (HSC) residing in the bone marrow. These cells mature into common lymphoid progenitors (CLPs) that migrate to the subcapsular region of the thymus (Doulatov et al., 2010; Iwasaki & Akashi, 2007). Here, they are considered double negative because they lack the expression of CD3, also called T cell receptor (TCR), and their typical co‐receptors CD4 and CD8. Due to the influence of the thymic microenvironment, the CLPs undergo a differentiation process where they gain expression of both CD4 and CD8 co‐receptors. Other surface markers such as CD25 and CD44 are also expressed during this time. Countless antigen‐specific TCRs are produced through a random process using somatic recombination. At this point, antigen specificities of T cells are utilized through the process of positive and negative selection in the thymus where immature T cells with low CD4 and CD8 expression are selected for their capability to recognize MHC class I and MHC class II complexes (Ashton‐Rickardt et al., 1994; Bevan, 1977). TCR affinity for the MHC class I molecules leads to differentiation into CD4‐CD8+ cytotoxic T cells whereas T cells showing affinity for the MHC class II complexes will differentiate into CD4+ CD8‐ helper T cells (Teh et al., 1988). Cytotoxic CD8+ T cells with a TCR that recognizes self‐peptides are eliminated through clonal deletion allowing for the generation of T cells that will be able to distinguish only non‐self antigens (Li et al., 2013; Schwartz, 1989). The end result is a naive precursor cytotoxic CD8+ T cell that possesses a unique antigen‐specific TCR to recognize peptides presented on MHC class I molecules and a CD8+ co‐receptor (Arosa et al., 2016). When any of the antigens presented on a MHC class I molecule are not self‐peptides, but foreign peptides or neo‐antigens, they will be recognized by a cytotoxic CD8+ T cell with a TCR specific for those antigens (Boon et al., 1994; Chen & Mellman, 2013). In the case of cancer cells, CD8+ T cells can recognize their foreign antigens and proceed to their clearance in a process described in seven steps according to the Cancer‐Immunity Cycle (Chen & Mellman, 2013). The first step is the release of tumour associated antigens, referred to as neo‐antigens, by the cancer cells. Neo‐antigens are expressed in these cells as a result of the accumulation of genetic alterations (Gilboa, 1999). Besides neo‐antigens, cancer cells can also be recognized by their expression of truly foreign antigens, for example those deriving from oncogenic viruses such as the Epstein‐Barr virus and human papilloma virus types 16 and 18 (Chang et al., 2017). Dendritic cells (DCs) perform the second step in the Cancer‐Immunity Cycle by capturing neo‐antigens to present them to naïve precursor CTLs (Popescu et al., 2014). DCs are immune cells from the innate immune system derived in the bone marrow but located in non‐lymphoid tissues during their immature period. Here, they continuously capture antigens from their environment using pattern recognition receptors such as toll like receptors (TLRs). When one of these antigens is a neo‐antigen from a tumour cell, the immature DC leaves the peripheral tissue and migrates through the draining lymph nodes to the spleen and the T‐cell zone of the subcapsular sinus of the lymph nodes where naive precursor CTLs are resided (Zhang & Bevan, 2011). The DC presents its captured antigens on its MHC class I molecules to the TCR of naive precursor CTLs (Kahan, 2003; Kapsenberg, 2003). This is the first contact of a cytotoxic CTL with its specific antigen and is called priming, the third step of Cancer‐Immunity Cycle (Popescu et al., 2014). After priming, naïve CTLs differentiate into effector cytotoxic CTLs. Important for the stability of the TCR interaction with the antigen‐MHC class I molecule complex is the binding of the CD8+ receptor to the constant part of the MHC class I molecule. During this process CD8+ T cells express multiple receptors to receive important survival and activation signals from ligands other than the MHC class I molecules (Rudd, 1996). The differentiation of the T cell is decided here by the binding of T‐cells CD28 receptor to CD80 (B7‐1) or CD86 (B7‐2/B70) ligands on the DC. However, this specific signal can be replaced by cytokines from helper CD4+ T cells (Kahan, 2003; Kapsenberg, 2003). The combination of multiple co‐stimulatory and inflammatory ligands determines a massive clonal expansion and the generation of a pool of effector cytotoxic CTLs which is composed of memory precursor cells and short‐lived effector cells, the latter of which destined to die after pathogens are cleared (Huster et al., 2004; Joshi et al., 2007; Kaech et al., 2003; Kalia & Sarkar, 2018; Kalia et al., 2010; Sarkar et al., 2008; Shrikant et al., 2010). At this stage, cytotoxic CTLs possess high migratory behaviour (Halle et al., 2017). They move from the lymphoid tissues to the blood stream to reach the peripheral target tissues where the cancer cells are located (Kalia & Sarkar, 2018). Migration to the tumour tissues and subsequent infiltration constitute the fourth and fifth step of the cycle, respectively.

### 3.2 Killing mechanisms

During the sixth step, effector CTLs recognize and bind to the cancer cell through the interaction between their TCRs and the specific neo‐antigen‐MHC class I complex on the cancer cell (Chen & Mellman, 2013). The activated effector cell could use three mechanisms to kill the malignant cell: The first is the secretion of effector cytokines, primarily TNF‐α and IFN‐γ (Popescu et al., 2014). The second mechanism is the release of cytotoxic granules, containing perforin and granzyme (Betts et al., 2003). The third mechanism of action is via Fas/FasL interactions. Effector CTLs express Fas ligands which bind to the Fas receptors expressed on the target cells. This interaction triggers signaling molecules that activate the caspase cascade. Specifically, the Fas‐associated death domain activates pro‐caspases 8 and 10 which in turn activate effector caspases 3, 6 and 7. Lastly, these activated caspases cleave death substrates resulting in apoptosis (Halle et al., 2017). Once the first cell is killed, cytotoxic CD8+ T cells move to a new target cell and kill again. In the seventh and last step of the Cancer‐Immunology Cycle, cancer cell elimination results in the release of additional tumour associated antigens and re‐activation of the whole cycle. After specific antigens are neutralized, the contraction phase occurs where the effector cells are eliminated to end the immune response (Kaech & Cui, 2012). An important component of this phase is the fratricide killing. Indeed, cytotoxic CD8+ T cells express Fas receptors themselves so that they can kill each other using their Fas ligand‐Fas receptor interactions. After this phase, about 5% to 10% of memory cells remain organized in small pools where they can last for years (Sprent & Surh, 2011). Some of these memory CD8+ T cells retain killing ability so that in case of a secondary infection or cancer cell appearance, they could respond quickly and efficiently (Böttcher et al., 2015; Gerlach et al., 2016; Kaech et al., 2002; Surh & Sprent, 2008; Wherry et al., 2003).

## 4 EXTRACELLULAR VESICLES

### 4.1 Definition

Most cell types, including those of the immune system, secrete lipid enclosed vesicles of different sizes and intracellular origin collectively known as extracellular vesicles (EVs). Exosomes are some of the most studied sub‐types of EVs originally described as ≈50 nm vesicles originating from multivesicular bodies (MVBs) (Johnstone et al., 1987; Lötvall et al., 2014). Similarly sized EVs have also been shown to form at the plasma membrane, as well as larger vesicles of around 1000 nm in diameter, typically referred to as microvesicles (Booth et al., 2006; Heijnen et al., 1999). Furthermore, cells can secrete large apoptotic bodies, adding to the pool of EVs found in the extracellular space (Hristov et al., 2004). Lately, some research groups announced the discovery of new classes of big‐sized EVs termed ‘oncosomes’ and ‘large oncosomes’. Even though their names would suggest a degree of similarity, these large EVs are considered different types of giant vesicles which were discovered in specific cancer contexts, glioblastoma multiforme and prostate cancer, respectively (Di Vizio et al., 2009; Meehan et al., 2016). They have demonstrated to shuttle functional oncogenic material such as tumour‐specific miRNAs or proteins to the recipient cells (Morello et al., 2013). Since there are several terms used to describe EVs, which are often used interchangeably, ISEV has attempted to address this issue through the publication of guidelines aimed at normalizing the terminology used to refer to EVs (Théry et al., 2018). They propose the term small EVs to refer to the vesicles less than 200 nm in diameter, and large EVs for vesicles greater than 200 nm.

### 4.2 Biogenesis and composition

Though small EVs have been reported to form directly from the plasma membrane, albeit from endosome‐like domains, small EV formation within the endosomal network is the much more characterized route of biogenesis (Booth et al., 2006; D'Souza‐Schorey & Schorey, 2018). Early endosomes are generated through inward budding of the plasma membrane, and these endosomes mature into late endosomes, the site for small EV, or exosome, formation (Stoorvogel et al., 1991). Through recruitment of proteins from the endosomal sorting complexes required for transport (ESCRT) to the endosome, 30–150 nm intraluminal vesicles (ILVs) bud into the endosome, thus creating MVBs (Hanson & Cashikar, 2012). These ILVs are composed of the membrane content of the endosome, as well as cytosolic content picked up during the budding of the vesicle. ILV formation in the absence of ESCRT machinery has also been described in a process dependent upon ceramide (Trajkovic et al., 2008). An apparatus involving multiple Rab proteins then transports the MVBs towards the plasma membrane (Li & Marlin, 2015). Finally, SNARE proteins on the MVB and plasma membrane interact, enabling fusion of the two membranes, thus releasing the vesicles out into the extracellular milieu as exosomes (Hessvik & Llorente, 2018). On the other hand, large EVs are formed at the plasma membrane, through outward budding into the extracellular space, giving birth to 1000 nm vesicles which are often called microvesicles (Tricarico et al., 2017). The budding process is dependent upon proteins involved in the endosomal pathway, including TSG101, ARF6 and ARRDC1. Like the smaller EVs, these larger vesicles form with both components of the cell membrane and cytosol. Big sized‐EVs such as oncosomes and large oncosomes shed from the plasma membrane of aggressive cancer cells. Given their high content in ARF6, they might share similar biogenesis mechanisms with large microvesicles (Di Vizio et al., 2012). Both large and small EVs act as mediators of cell‐cell communication, though there may be some differences in the cargo they are able to carry and deliver to cell, perhaps due to the size disparity between the vesicles, or distinct mechanisms of cargo loading at the MVB versus the plasma membrane (Kanada et al., 2015; Yáñez‐Mó et al., 2015).

An EV is composed of a lipid bilayer coated with membrane bound proteins, with a number of molecules contained within the vesicle lumen. Amongst the proteins expressed by EVs are tetraspanins, notably CD9, CD63, and CD81, which are typically detected on the surface of EVs (Escola et al., 1998; Théry et al., 1999). The ESCRT‐associated proteins ALIX and TSG101 are also expressed in small EVs, and are often used as markers for MVB‐derived exosomes (Théry et al., 2001). Besides the proteins used as general EV markers, EVs also carry proteins specific to their cell of origin reflecting the state of the parental cell. For example, B16‐F10 metastatic melanoma cells express MET to a far greater extent than a non‐metastatic counterpart, a situation also reflected in MET expression on the respective EV populations (Peinado et al., 2012). Much like protein content, lipid content too can vary in EVs depending on their secreting cell, though some lipids are generally enriched in EVs versus cells, such as cholesterol, phosphatidylserine and sphingomyelin (Record et al., 2014; Skotland et al., 2019). The presence of nucleic acids, particularly RNA, in EVs is also well established (Turchinovich et al., 2019). Elucidating the role these cargoes play in EV‐mediated cellular signaling is of high interest to gain a greater understanding of the function of EVs in health and disease.

## 5 EFFECTS OF NK‐DERIVED EXTRACELLULAR VESICLES ON TUMOUR CELLS

**FIGURE 1:**
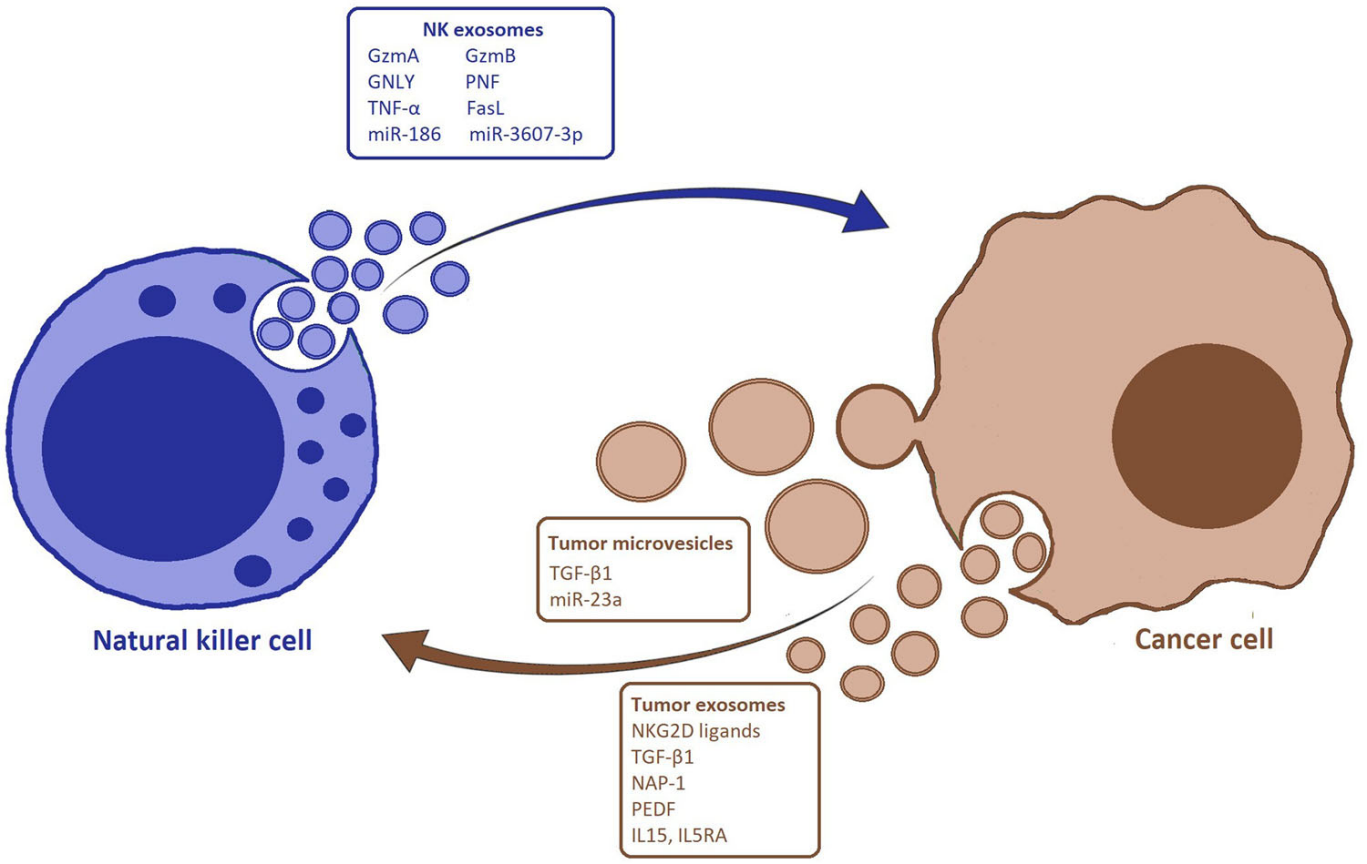
Schematic representation of extracellular vesicles‐mediated crosstalk between Natural killer cells (NKs) and cancer cells. [NKs and cancer cells communicate through extracellular vesicles (EVs), such as exosomes and microvescicles. Exosomes are generated in the late endosomes when small vesicles are internalized, thus creating multivesicular bodies which then fuse with the cellular membrane and deliver their exosomal content in the extracellular space. Microvesicles are larger EVs produced by outward budding of the plasma membrane. EVs molecular cargo includes different types of biomolecules, including proteins and microRNAs, that could be exchanged leading to functional effects on both cells]

NK cells can use various molecules contained in their EVs to kill their target cells (Figure 1, Table 1). A large share of this repertoire is represented by cytolytic proteins that seem to contribute collectively to the resulting cytotoxicity effect. An article by Wu et al. described the presence of multiple cytotoxic proteins such as granzyme A (GzmA), granzyme B (GzmB), granulysin, (GNLY), and perforin (PFN) in NK‐derived EVs acting on various cell death pathways without a dominant killing factor. They showed damage of mitochondria in neuroblastoma and acute lymphoblastic leukaemia (ALL) cells exposed to NK‐derived EVs with subsequent activation of caspase‐dependent apoptotic mechanisms. Targeted cancer cells also displayed increased degradation of proteins associated with DNA assembly (an event indicative of caspase‐independent death pathways) and ER‐stress induction leading to necroptosis (Wu et al., 2019). Tumour necrosis‐alpha (TNF‐α) and Fas‐ligand (FasL) are also contained inside EVs released by NK cells. Zhu et al. reported their presence in exosomes generated by the cell line NK92 to treat melanoma cells. The authors reported the activation of intrinsic and extrinsic apoptotic markers in targeted melanoma cells that was coupled with reduced cell proliferation and tumour burden in in vitro and in vivo experiments (Zhu et al., 2017). Analyzing the proteome composition through mass spectrometry, Korenevskij et al. found molecules of GrzmA in microvesicles released by NK92 cells (Korenevskii et al., 2018). These data suggest that NK cells can spread their cytotoxic proteins through different EVs further boosting the vast killing potential these cells have to fight cancer cells. Interestingly, proteins are not the only antitumoral molecules that could be found inside NK EVs. A study by Neviani et al. evidenced the role of the tumour suppressor miR‐186‐5p contained in NK‐derived exosomes and its cytotoxic effect on neuroblastoma cells (Neviani et al., 2019). The authors noticed that low levels of miR‐186 in patients with high risk of developing neuroblastoma corresponded with reduced expression of the two NK activation markers NKG2D and DNAM‐1 which was caused by an intense activation of the TGFβ signaling pathway. Mir‐186 was detected in exosomes produced by TGFβ‐activated NK and it was found to inhibit key components of the TGFβ pathway such as TGFβR‐1 and ‐2, MYCN, and AURKA. Based on these observations, they delivered miR‐186 in NK cells previously activated by TGFβ showing that it was able to restore NK‐exosomes cytotoxicity and limit immunoevasion effects related to the TGFβ treatment. Additionally, they administered miR‐186 to neuroblastoma cells and in vivo orthotopic models noticing that it led to impaired tumour growth. Mir 3607‐3p constitutes another example of biologically active miRNA present in NK‐derived EVs. This miRNA is present at low levels in patients affected by pancreatic cancer and is often associated with a poor prognosis. Sun et al. found higher levels of miR 3607‐3p in NK‐derived EVs and reported that it could be transmitted from NK cells to pancreatic cancer cells in co‐culture where it inhibits migration, proliferation and invasive capacity of the tumour cells, presumably regulating its candidate target IL‐26. (Sun et al., 2019). They also described reduced tumour burden and metastasis formation in tumour‐bearing mice when treated with NK‐pancreatic cancer cells co‐inoculation, even though they did not explore any EV involvement in these effects. The cytolytic potency of NK‐derived EVs can be further enhanced by priming with IL‐15. These are the results of a study conducted by Zhu et al. where EVs harvested from NK cells previously treated with IL‐15 demonstrated a superior antitumour potency coupled with augmented expression of cytotoxicity‐associated molecules when compared with untreated NK‐EVs. NK‐EVs primed with IL‐15 also produced a stronger inhibition of glioblastoma xenograft growth with negligible toxicity on the mice (Zhu et al., 2019). Even if not strictly attributed to NK cells, results from a study conducted by Lee et al. reported a protective role played by innate EVs in the plasma of melanoma patients after surgical resection (Lee et al., 2019). They measured higher levels of plasma EVs accompanied by a stronger inhibitory effect on tumour cells proliferation in patients post‐tumour excision compared to patients affected by metastatic melanoma. At a molecular level, plasma EVs delivered miR‐34a to melanoma cells which caused down‐regulation of β‐catenin pathway and block of the uncontrolled tumour proliferation. These findings lead to hypothesize that primary melanoma could induce the generation of a pool of innate plasma EVs able to check residual tumour cells after removal of the primary mass, thereby preventing possible relapses. Also, they point to a new mechanism of antitumour activity where immune EVs tend to inhibit rather than kill tumour cells. Though encouraging, these results will need to be further confirmed by additional in vivo studies. Considering all these data, NK‐derived EVs express a great variety of cytotoxic potential which could be helpful for clinical application.

**TABLE 1:** Signature of natural killer extracellular vesicles (EVs) and their effects on tumour cells

Natural killer exosomes (NKEXs)						
EVs signature		Tumour model	Mechanisms of action	Effects on tumour cells	References	Type of study
Biomolecules	Localization					
Granzyme A (GzmA), Granzyme B (GzmB), Granulysin, (GNLY), Perforin (PFN)	Cytoplasm	Neuroblastoma and acute lymphoblastic leukaemia (ALL) cells	Damage of mitochondria, promoting degradation of proteins associated with DNA assembly, induction of ER‐stress	Activation of caspase‐dependent apoptotic mechanisms; Necroptosis	(Wu et al., 2019)	In vitro
Tumour necrosis‐alpha (TNF‐α) Fas‐ligand (FasL)	Cytoplasm Membrane	Melanoma	Activation of intrinsic and extrinsic apoptotic markers	Reduction of cell proliferation and tumour burden	(Zhu et al., 2017)	In vitro + in vivo
miR‐186	Cytoplasm	Neuroblastoma cells	Downregulation of TGFBR1 and TGFBR2	Alteration of immunoevasion	(Neviani et al., 2019)	In vitro + in vivo
miR 3607‐3p	Cytoplasm	Pancreatic cancer cell	Predicted targeting IL‐26	Inhibition of migration, proliferation and invasive capacity	(Sun et al., 2019)	In vitro, + human material

## 6 EFFECTS OF EVS PRODUCED BY CYTOTOXIC CD8+ T CELLS ON TUMOUR CELLS

**FIGURE 2:**
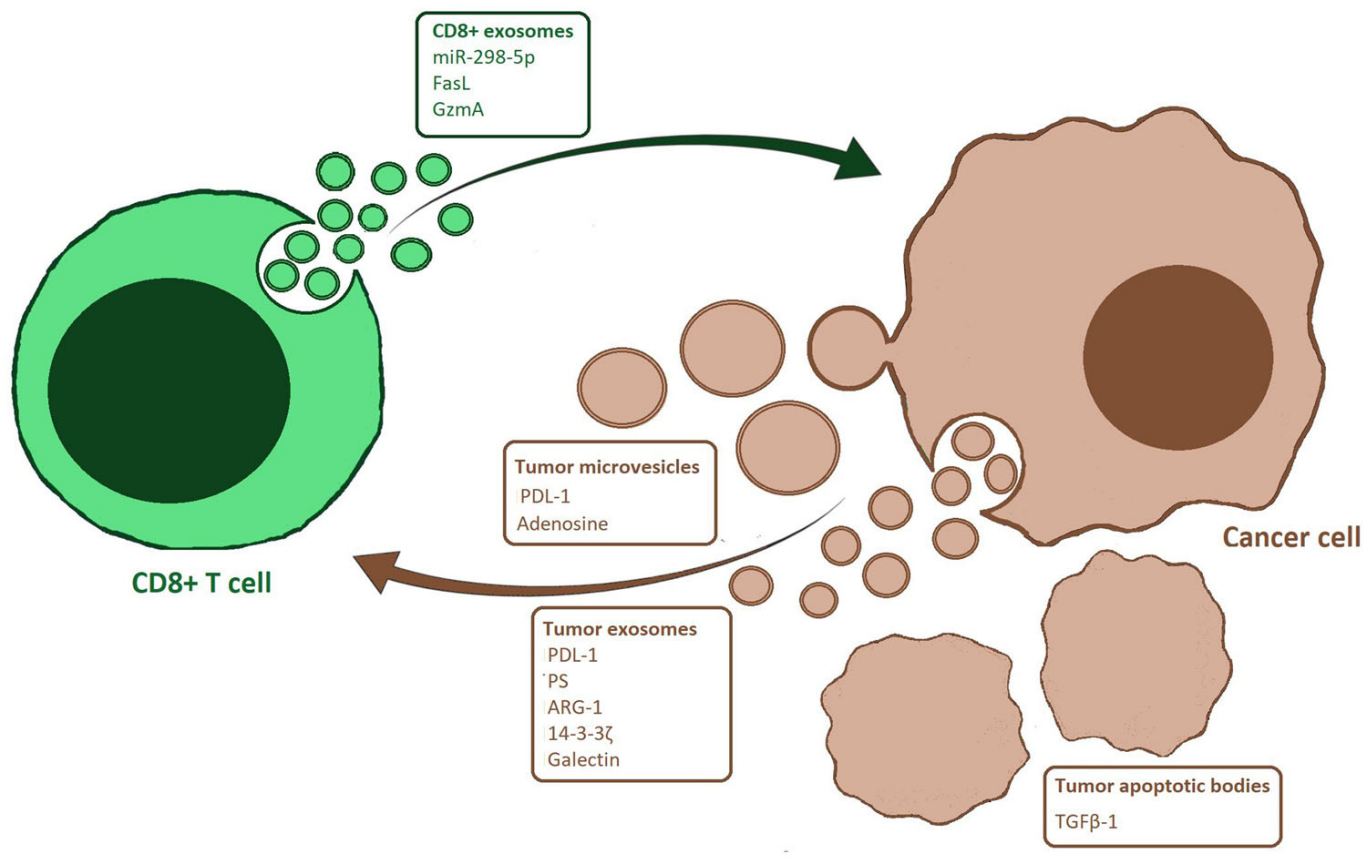
Schematic representation of extracellular vesicle‐mediated crosstalk between CD8+ T cells and tumour cells. [CD8+ T cells and cancer cells communicate through extracellular vesicles (EVs), such as exosomes, microvescicles and apoptotic bodies. Adding to traditional EVs, apoptotic bodies are large fragments of cellular material shedding from tumour cells which could be released in the extracellular milieu]

Cytotoxic CD8+ T lymphocytes (CTLs) together with CD4+ T helper (Th) cells constitute the armed division of the adaptive immune response. Generally, they recognize and kill tumour cells by direct cell‐to‐cell contact carrying out the significant task of eliminating the tumour threat as first responders (Andersen et al., 2006). Although still few, some interesting reports in the last years described an increasingly important role for CTL‐derived EVs in the tumour microenvironment (Table 2). A list of the most recent molecules exchanged trough EVs between CTLs and cancer cells is displayed in Figure 2. Pyroptosis has been described as an additional new mechanism of tumour killing adopted by cytotoxic T cells. It consists of a necrotic cell death conveyed by the activation of gasdermin‐B (GSDMB) on the surface of the tumour cell. GSDMB, abundantly expressed in the upper gastrointestinal tract epithelium and in some cancer cell lines (Hergueta‐Redondo et al., 2014; Saeki et al., 2009), is cleaved and consequently activated after the release of Granzyme A from CTLs. When it becomes activated, GSDMB mediates pores formation in tumour cells which are ultimately swollen and destroyed (Zhou et al., 2020). CTLs showed to be effective also in the killing of tumour stromal cells. According to the study of Seo et al., EVs generated by CTLs in healthy mice were taken up in the tumour stroma by different cells such as mesenchymal‐derived cells (MSCs) and cancer associated fibroblasts (CAFs), leading to reduced invasive and metastatic capabilities. miR‐298‐5p contained in CTL‐derived EVs resulted to be involved in the depletion of mesenchymal tumour cells even if the authors concluded that its mechanism of action remains to be investigated (Seo et al., 2018). These remarkable findings reveal how CD8+ T cells anti‐tumoral potential is not limited to a direct effect on tumour cells but can include the stromal components, ‘allies’ of the tumour cells to create a suitable microenvironment. As previously reported also for NK cells, some data claimed a pro‐tumoral effect attributed to CTL‐derived EVs. The paper by Cai et al. documented that melanoma and lung cancer cells exposed to CD8+ exosomes secreted by T cells purified from tumour‐bearing mice tended to metastatize more frequently compared to exosomes isolated from healthy mice. The study showed evidence that exosomes released by CTLs contained FasL but led to increased invasive activity from tumour cells in vitro and in vivo through the upregulation of MMP9. Specifically, activated T‐cell exosomes triggered accumulation of c‐FLIP, a known inhibitor of apoptotic death, in tumour cells thus stimulating the activation of ERK and NF‐κB pathways which in turn contributed to increased expression of MMP9 (Cai et al., 2012). When exposed to the tumour microenvironment, CTLs might become corrupted entering a particular state of dysfunctional activity called exhaustion where they are not able to efficiently perform their effector functions. Exhausted T cells have been demonstrated to release exosomes capable of altering proliferation and cytotoxic performance from healthy T cells (Wang et al., 2019). However, the communication between CTLs and other immune cells is often advantageous in terms of anti‐tumoral activity. CTLs can produce EVs that can be internalized by naïve bystander T cells stimulating them to contribute to the production of cytokines or further triggering weakly activated T cells to recognize and destroy tumour cells, even without antigen presence (Li et al., 2017). Equally, exosomes from high‐affinity CTLs were able to boost proliferation and cytokine secretion in low‐affinity T cells (Wu et al., 2019). These results prove that EVs from CTLs facilitate interactions between these cell types and significantly contribute to the generation of an efficient anti‐tumoral response.

**TABLE 2:** Signature of CD8+ T cells extracellular vesicles (EVs) and their effects on tumour cells

CD8+T cells exosomes (CD8+ EXs)						
EVs signature		Tumour model	Mechanisms of action	Effects on Tumour cells	References	Type of study
Biomolecules	Localization					
miR‐298–5p	Cytoplasm	Tumour stromal cells (MSCs, CAFs)	Promotion of apoptotic depletion	Reduction of invasive and metastatic capabilities.	(Seo et al., 2018)	In vitro + in vivo
Fas‐ligand (FasL)	Membrane	Melanoma and Lung cancer cells	Accumulation of c‐FLIP; promotion of ERK and NF‐kB pathways and MMP9 expression	Increase of invasive activity	(Cai et al., 2012)	In vitro + in vivo
Granzyme A (GzmA)	Cytoplasm	Gasdermin B (GSDMB)‐positive cells	Cleavage of GSDMB	Pyroptosis	(Zhou et al., 2020)	In vitro + in vivo

## 7 TUMOUR‐DERIVED EV EFFECTS ON NK CELLS

### 7.1 Tumour exosomes (TEXs)

The release of exosomes has lately emerged as one of the most effective and potent fighting weapons that tumour cells use to hinder NK cell antitumour activity (Table 3). The main effect caused by TEXs interaction with NK cells is the downregulation of the NK surface receptors, particularly NKG2D, a central receptor for NK cytotoxicity (Jamieson et al., 2002). Tumour cells are able to secrete NKG2D ligands, such as MHC class I chain‐related proteins (MICA and B) or UL‐16 binding proteins (ULBP), expressed on the surface of their exosomes which, upon interaction with NKG2D receptors on NK cells surface, determine their downregulation resulting in a general dysfunction of the immune cell (Ashiru et al., 2010). Many studies reported this effect among different types of cancer suggesting that it is a common mechanism for immunoevasion (Labani‐Motlagh et al., 2016; Ludwig et al., 2017; Lundholm et al., 2014). In a study by Labani‐Mothlag et al, the authors showed specific reduction of NKG2D expression on NK cell surface compared to another important cytotoxicity receptor such as DNAM‐1 when the cells were exposed to ovarian cancer exosomes. The selective expression of NKG2D ligands on tumour exosomes and the inhibition of its receptors on the NK cells was accompanied by a lesser expression of DNAM‐1 ligands and unaffected DNAM‐1 receptor expression suggesting that NKG2D might be a more prominent target for tumour cells (Labani‐Motlagh et al., 2016).

**TABLE 3:** Signature of tumour extracellular vesicles (EVs) and their effects on natural killer cells

EVs signature		Tumour EVs origin	Mechanisms of action	Effects on NK cells	References	Type of study
Biomolecules	Localization					
Tumour exosomes (TEXs)						
NKG2D ligands (MICA and B, ULBP)	Surface	Cervical cancer, Liver cancer and Melanoma cells, Prostate cancer, Ovarian cancer, HNC with active disease (AD)	Downregulation of the activating receptor NKG2D	Dysfunction of NK cells	(Ashiru et al., 2010), (Lundholm, 2014), (Labani‐Mothlag, 2016), (Ludwig et al., 2017)	In vitro In vitro + human material In vitro + human material In vitro + human material
TGF‐β1	Cytoplasm	Clear cell renal cell carcinoma (ccRCC), Pancreatic cancer, Acute myelogenous leukemia	Phosphorylation and activation of Smad‐2/3 proteins; Downregulation of NKG2D, CD107a, CD71, and CD98; Inhibition of production of cytokines (TNF‐alpha, IFN‐y)	Alterations of glucose uptake, cytokine production, cytotoxic and anti‐tumour activity	(Xia et al., 2017) (Zhao et al., 2019) (Whiteside, 2013)	In vitro + human material In vitro In vitro + human material
Immunosuppressive proteins (including TGF‐ß)	Cytoplasm	Acute myeloid leukemia	Downregulation of chemokine (CXCR4, CCL3, CCL4, and CCL5); Downregulation of NKG2D; increase of adenosine, inosine and hypoxanthine	Inhibition of cytotoxicity, differentiation, proliferation and leukemia‐directed migration	(Hong et al., 2017)	In vitro + human material
NF‐kB‐activated kinase‐associated protein 1 (NAP‐1)	Cytoplasm	Oral cancer	Regulation of the interferon regulatory factor 3 (IRF‐3); activation of gene expression (type I interferon (IFN), chemokine ligand (CXCL))	Stimulation of proliferation and cytotoxic potential	(Wang et al., 2018)	In vitro
Pigment epithelium‐derived factor (PEDF)	Cytoplasm	Non‐metastatic Melanoma	N/A	Recruitment of NK cells to inhibit lungs colonization	(Plebanek et al., 2017)	In vitro + in in vivo + human material
IL15, IL15RA	Surface	Senescence‐induced myeloma cells	N/A	Promotion of proliferation and activation	(Borrelli et al., 2018)	In vitro + human material
Lentiviral shRNA for TGF‐β1 (engineered TEXs)	Cytoplasm	Leukemia cells	N/A	Increase of cytolysis activity	(Huang et al, 2017)	In vitro + in vivo
Tumour microvesicles						
TGF‐β1	Cytoplasm	Leukemic blasts, Hypoxic tumours	Downregulation of NKG2D and phosphorylation of Smad 1, 5 and 8	Inhibition of NK cells activity	(Szczepanski et al., 2011)	In vitro + human material
miR‐23a	Cytoplasm	Hypoxic tumours	Target CD107a expression	Reduction of degranulation and cytolysis	(Berchem et al., 2016)	In vitro

N/A = the paper did not propose or show the molecular mechanism of action.

It should be noted though that downregulation of NKG2D receptor could be partially dependent on NKG2D ligands delivered by tumour exosomes (Lundholm et al., 2014).

The TGF‐β/Smad signaling pathway has been well characterized in NK cell functional suppression by TEXs. In clear cell renal cell carcinoma (ccRCC), exosomes generated by the tumour cells displayed high levels of TGF‐β1 that caused sustained phosphorylation and subsequent activation of Smad‐2/3 proteins in NK cells leading to a weakened degranulation and killing ability toward target cells after treatment with ccRCC TEXs (Xia et al., 2017). The partial functional recovery observed in NK cells activity after exposure to ccRCC‐exosomes containing siRNA‐silenced TGF‐β1 confirmed the immunosuppressive role for TGF‐β1 in this context.

Zhao et al. highlighted the same inhibitory effect of exosomal TGF‐β1 on NK cells when the latter were treated with pancreatic cancer exosomes. The authors proposed a mechanism where TGF‐β1 delivered by pancreatic TEXs binds to TGF‐β receptors I and II on the NK cell surface triggering phosphorylation of Smad2/3. Once activated, Smad2/3 enter the nucleus and regulate the expression of various molecules including NKG2D, CD71, CD98 with the ultimate effects of altering glucose uptake, cytokine production, and tumour killing (Zhao et al., 2019). Despite their relevance, these data come from in vitro experiments and need to be strengthened by in vivo observations. A further advancing step has been made by the work of Whiteside et al. where they report the presence of TGF‐β1‐loaded exosomes in the sera of patients affected by acute myelogenous leukaemia (AML). Their findings reveal a characteristic molecular profile for TEXs isolated from AML patients and substantiated the role of the TGF‐β1‐Smad2/3 pathway in NKG2D downregulation, implying that the activation of this pathway in NK cells is, at least partially, responsible for the lowered anti‐tumour activity expressed by these cells in AML (Whiteside, 2013).

On the other side, leukaemia cells treated with a lentiviral shRNA for TGF‐β1 produced lesser amounts of TGF‐β1 in their exosomes. When exposed to leukaemia exosomes containing a silenced version of TGF‐β1, NK cells exhibited a stronger and more efficient cytolysis compared to the same cells exposed to unmodified leukaemia exosomes (Huang et al., 2017). These results stressed the central role played by TGF‐β1 enclosed in TEXs in the delivery of a tolerogenic signal aimed at NK cells.

But NKG2D downregulation operated through the activation of the TGF‐β1‐Smad2/3 pathway activation is not the only mechanism used by TEXs to unleash their immunosuppressive potential. In AML, NK cells incubated with leukaemia‐derived exosomes (LEXs) showed significant downregulation of a group of chemokine comprising CXCR4, CCL3, CCL4, and CCL5 involved in cell migration. A marked increase of adenosine production by NK cells was also detected together with reduced migration capacity leading to the conclusion that LEXs exerted a sort of ‘paralyzing’ effect on NK cells acting directly on the cells and, at the same time, forcing them to stop their movements autonomously. Also, NK cells didn't need to necessarily internalize LEXs to be subjected to their effect, a proof of the multiple ways of interaction that LEXs possess to be effective on their target cells (Hong et al., 2017). An innovative study by Katsiougiannis et al. provided a novel mechanism of immunoevasion showed by TEXs secreted from distal tumours. The authors used murine models of pancreatic cancer to investigate the effects of TEXs on peripheral NK cells through the recruitment of salivary gland‐derived exosomes. They demonstrated that in tumour‐bearing mice TEXs released by the primary tumour were able to alter the transcriptome of salivary exosomes which significantly lowered peripheral NK cells cytotoxicity when the saliva of tumour‐bearing animals was transferred to naïve mice by oral gavage. TEXs’ responsibility for this effect was proved by the observation of the ablation of NK cells impairment when exosome biogenesis was suppressed in tumour‐bearing mice and the direct uptake of the transformed salivary exosomes from NK cells in naïve mice (Katsiougiannis et al., 2017).

Alternatively, other studies remarked a positive role for TEXs in the regulation of NK cells activity. Oral cancer‐exosomes (OCEXs) were found to stimulate proliferation and cytotoxic potential when co‐cultured with NK cells. Specifically, OCEXs contained high levels of NF‐kB‐activated kinase‐associated protein 1 (NAP‐1), an important upstream regulator of the interferon regulatory factor 3 (IRF‐3), which activates the expression of the type I interferon (IFN) gene and some chemokine ligand (CXCL) genes (Wang et al., 2018). Using in vitro and in vivo experiments, Plebanek et al. postulated an immunostimulatory effect attributed to the TEXs. They utilized exosomes isolated from poorly metastatic melanoma cell lines and patients with non‐recurring melanomas and pretreated mice which were then injected with syngeneic melanoma cells, noticing that the treatment resulted in inhibition of lung colonization compared to the use of aggressive melanoma cells. In this study, TEXs from non‐metastatic cells appeared to be enriched in pigment epithelium‐derived factor (PEDF), strongly attracting patrolling monocytes to the pre‐metastatic niche with recruitment of NK cells and tumour cells clearance (Plebanek et al., 2017). The description of stimulating signals occasionally produced by TEXs led some researchers to try to take advantage of them for clinical purpose. This is the case of the study by Borrelli et al. where the triggering of a senescent status in myeloma cells through low dosage of chemotherapeutic drugs promoted NK cell proliferation and activation after co‐incubation with the tumour cells. Senescence‐induced myeloma cells expressed higher levels of IL‐15 on the surface and in their exosomes which transferred it to neighbouring NK cells potentiating their functionality (Borrelli et al., 2018). These data depict a picture of great complexity in the understanding of TEXs immunoregulatory effects on NK cells and demand further investigation to better grasp the meaning of this double role.

### 7.2 Microvesicles

Tumour cells shed microvesicles in their microenvironment as an additive mechanism to educate NK cells. Even though the amount of research conducted on this field is limited compared to the hot field of exosomes, recent articles came out revealing interesting findings (Table 3). TGF‐β1 as key inhibitor of NK cells activity was found to be highly loaded in tumour‐derived microvesicles too, confirming its usage by tumour exosomes and microvesicles alike as a common mechanism for immunoevasion. Szczepanski et al. reported high levels of TGF‐β1 in leukemic blasts‐derived microvesicles isolated from the sera of patients with AML. After incubation with AML‐derived microvesicles, NK cells exhibited functional alterations related to NKG2D downregulation and sustained phosphorylation of Smad 1, 5 and 8. This effect was not seen when NK cells were exposed to microvesicles obtained from the sera of healthy donors and the use of TGF‐β1‐neutralizing antibodies restored normal NK cells functionality in the co‐cultures, indicating that the observed results could be specifically attributed to the immunosuppressive signaling by TGF‐β1 enclosed in tumour‐shed microvesicles (Szczepanski et al., 2011). TGF‐β1 involvement has also been shown in hypoxic tumours. Microvesicles shed by different hypoxic tumours resulted enriched in TGF‐β1 which was then delivered to NK cells causing reduced antitumour activity through inhibition of NKG2D expression. Furthermore, tumour microvesicles were able to shuttle miR‐23a in NK cells where it directly targeted CD107a expression, greatly reducing NK degranulation and cytolysis (Berchem et al., 2016).

## 8 EFFECTS OF TUMOUR‐DERIVED EVS ON CD8+ T CELLS

### 8.1 Tumour exosomes (TEXs)

Tumour cells employ EVs as efficient weapons in their defense against immune cells, including CD8+ T cells (Figure 2, Table 4). In this context, the PD‐L1/PD‐1 axis plays a central role and it is believed to be one of the major molecular interaction to take place when tumour cells and CD8+ T cell come into contact. Programmed death ligand‐1 (PD‐L1) is the natural ligand of programmed cell death protein‐1 (PD‐1), an inhibitory checkpoint molecule expressed on T‐cells surface that stop cell activation when it is bound to PD‐L1. PD‐L1 has been found on the surface of many cells belonging to diverse tumour types but ultimately several studies discovered its presence on tumour exosomes surfaces too. Kim et al. noticed that PD‐L1 expression on the surface of exosomes produced by non‐small cells lung cancer (NSCLC) greatly resembled PD‐L1 expression on the cellular surface in terms of abundance. NSCLC exosomal PD‐L1 was found to suppress IFN‐γ secretion in CD8+ T cells, halting their proliferation and inducing apoptosis (Kim et al., 2019). Similarly, metastatic melanoma cells were shown to generate large amounts of exosomes displaying PD‐L1 on their surface that were able to counteract anti‐tumour immunity. Exosomal PD‐L1 expression resulted increased after IFN‐γ stimulation and metastatic melanoma exosomes appeared to be more prone to engage PD‐1 expressing CD8+ T cells which are the larger contributors to IFN‐γ secretion (Chen et al., 2018). These data would likely reflect a situation where tumour cells could take advantage of the increased IFN‐γ production by the T cells in order to potentiate their exosomal PD‐L1 expression and vigorously fight back. The immunoinhibitory potential of exosomal PD‐L1 demonstrated far‐reaching capabilities. In a study by Poggio et al., the authors presented results showing that PD‐L1 expressed on TEXs surface can inactivate CD8+ T cells in the draining lymph nodes. This means that tumour exosomal PD‐L1 can travel through the body to deliver its anti‐immunity effects, blocking T cells activity not only at the tumour site but also systemically. Indeed, the authors showed that mice injected with tumour cell lines unable to secrete exosomes and subsequently treated with PD‐L1‐expressing TEXs exhibited tumour formations even at distance from the injection site compared to mice injected with the same cells but treated with PD‐L1 null TEXs (Poggio et al., 2019). PD‐L1 could also be found free in its soluble form in the circulation. The soluble PD‐L1 (sPD‐L1) has just begun to be investigated but it already revealed different characteristics compared to its membrane‐bound form. After its cleavage from plasmatic or exosomal membranes, sPD‐L1 demonstrated a similar immunosuppressive role to its membranous counterpart being able to reduce killing capacity or inducing apoptotic signals in CD8+ T‐cells (Orme et al., 2020). However, a paper by Ng et al. documented the existence of an alternative spliced variant of sPD‐L1 that actually acts as an antagonist to its same receptor. The authors discovered that a new variant called CD274‐L2A was generated through exonisation of an intronic LINE element in the CD274 gene sequence which led to the translation of a protein deprived of its transmembrane domain but still able to bind PD‐1. Considered the major source of human sPD‐L1, CD274‐L2A determined slower growth of the colon adenocarcinoma murine cell line MCA‐38 when these cells were injected in immunocompetent mice (Ng et al., 2019). This effect induced the authors to believe that CD274‐L2A can be supportive of T‐cell anti‐tumour immunity, displaying an opposite behaviour to its membrane form. Given the paucity of observations in animal models, not much could be said about the immunoregulatory roles of sPD‐L1. Still, the contrasting behaviour highlighted by some studies points to the existence of potential differences compared to the more consistent immunosuppressive role expressed by its exosomal form. The presence of EV‐associated PD‐L1 has been discovered in non‐tumour cells too. By using in vitro models supported by mice experiments, Lee‐Chang et al. showed how myeloid‐derived suppressor cells (MDSC) infiltrating the brain produce EVs containing PD‐L1 which are subsequently acquired by regulatory B‐cells (Bregs) in glioblastoma multiforme (Lee‐Chang et al., 2019). Exposed to vesicular PD‐L1, Bregs are shifted towards an immunosuppressive phenotype that leads to a vigorous CD8+ T‐cell inhibition. Fibroblasts are also capable of secrete PD‐L1 inside their EVs. When stimulated by TGF‐β, human lung fibroblasts demonstrated to release EVs containing PD‐L1 in order to halt T‐cell activation and facilitate their migration (Kang et al., 2019). Given the key role of fibroblasts in the tumour microenvironment, it might be argued that cancer cells might also employ this alternative process to defend themselves from cytotoxic T cells activity. These findings point to an indirect effect of the tumour cells where they can take advantage of surrounding cells and use their EVs arsenal to inactivate T‐cells anti‐tumour response. Another immunoregulatory protein expressed on TEXs’ surface is phosphatidylserine (PS). TEXs originated from ascites fluid or solid tissue from ovarian cancer patients proved to trigger a quick and reversible arrest in CD8+ T cells, mainly by blocking the T‐cell receptor signaling pathways. This effect was related to the expression of PS on the outer layer of the exosomal membrane as evidenced by the abrogation of T cells arrest after depletion of PS expression on TEXs (Kelleher jr et al., 2015). An additional study on ovarian cancer patients revealed the presence of TEXs from ascites and plasma enriched in arginase‐1 (ARG‐1). The study found that ARG‐1+ EVs generated by ovarian cancer cells induced proliferation arrest of T‐cells through the inhibition of their signaling activation keypoints CD3ζ and CD3ε (Czystowska‐Kuzmicz et al., 2019) . In hepatocellular carcinoma (HCC), tumour cells were able to release exosomes loaded with the protein 14‐3‐3ζ towards infiltrating CD8+ T cells. Once internalized, 14‐3‐3ζ led to increased levels of T cells exhaustion markers such as PD‐1 and TIM‐3 determining the onset of an anergy status which related to T cell inactivation and proliferation halt (Wang et al., 2018). TEXs can also turn functional CD8+ T cells into self‐suppressed cells through the induction of a dysfunctional phenotype characterized by the loss of the CD27 and CD28 markers. Maybruck et al. observed the emergence of this phenotype in CD8+ T cells previously exposed to TEXs originated from head and neck cancer cells. They postulated that galectin, contained in TEXs, could be partly responsible for the inhibition of the previously mentioned markers and the weakened IFN‐γ production in exposed T cells to ultimately favour the appearance of the suppressor phenotype (Maybruck et al., 2017). The transcriptional landscape of cytotoxic T cells could be also significantly impacted by the effects of TEXs. Melanoma cells‐derived exosomes demonstrated to modify the transcript content of cytotoxic T cells in vitro, profoundly altering mitochondrial respiration pathways and upregulating the expression of genes belonging to the Notch signaling family (Bland et al., 2018). Based on these findings, it could be said that tumour cells are able to deliver a plethora of immunoregulatory molecules through their exosomes; for this reason, research must be encouraged in this direction to widen our understanding of their mechanisms of action and subsequently devise diversified therapeutic options.

**TABLE 4:** Signature of tumour extracellular vesicles (EVs) and their effects on CD8+ T cells

EVs signature		Tumour EVs origin	Mechanisms of action	Effects on CD8+ T cells	References	Type of study
Biomolecules	Localization					
Tumour exosomes (TEXs)						
PD‐L1	Surface	Non‐small cells lung cancer, Metastatic melanoma cells, Prostate cancer cells	Suppression of IFN‐γ secretion	Inhibition of proliferation, anti‐tumour activity; Induction of apoptosis	(Kim et al., 2019) (Chen et al., 2018) (Poggio et al., 2019)	In vitro + in vivo + human material In vitro + in vivo + human material In vitro + in vivo
Phosphatidylserine (PS).	Surface	Ovarian cancer	Block of the T‐cell receptor signaling pathways	Quick and reversible arrest of CD8+ T cells	(Kelleher et al. 2015)	In vitro + human material
Arginase‐1 (ARG‐1)	Cytoplasm	Ovarian cancer	Inhibition of signaling activation CD3ζ and CD3ε	Induction of proliferation arrest of T‐cells	(Czystowska‐Kuzmicz et al., 2019)	In vitro + in vivo + human material
14‐3‐3ζ	Cytoplasm	Hepatocellular carcinoma (HCC)	Increase of PD‐1 and TIM‐3	Block of proliferation and inactivation of CD8+ T cells	(Wang et al., 2018)	In vitro + human material
Galectin	Surface	Head and neck cancer cells.	Loss of the CD27 and CD28, Inhibition of IFN‐γ production	Activation of self‐suppressor phenotype	(Maybruck et al., 2017)	In vitro + human material
Non‐exosomal tumour EVs						
Adenosine	Cytoplasm	Breast cancer	Activation of adenosine receptors	Inhibition if perforin secretion	(Tadokoro et al., 2020)	In vitro + human material
PD‐L1	Surface	Breast cancer		Inhibition of anti‐tumour activity	(Timaner et al., 2020)	In vitro + in vivo
TGFβ‐1	Surface of irradiated apoptotic bodies	Mouse thymic lymphoma	Activation of the transcriptional regulator NF‐AT	Trigger of anergic status	(Xie et al., 2009)	In vitro + in vivo

### 8.2 Ectosomes/non‐exosomal EVs

Even if exosomes still attract the major part of the research in the field, non exosomal EVs have been reported to be part of the evading strategies used by tumour cells against the immune system. Recently, innovative research has been produced regarding the role of large non‐exosomal EVs (Table 4). Tadokoro et al. published an interesting paper which demonstrates how breast cancer cells can ‘sacrifice’ their EVs in order to impair cytotoxic T cell functionality. T cells employ perforin to create pores in the tumour EVs to destroy them but, in doing so, they also allow the release of tumour EVs’ content in the extracellular space. The authors noticed that one of the most abundant metabolite contained in tumour EVs was adenosine which bound to specific adenosine receptors on T cell surface after EVs’ bursting. The activation of adenosine receptors was followed by a fall in perforin secretion in the T cell, leading the authors to conclude that tumours could use EVs not only for offensive purposes but also as expendable tools to render immune cells harmless (Tadokoro et al., 2020). Other studies pointed to the effect of radiotherapy on the generation of tumour membrane blebs containing molecules with immunomodulatory properties. In breast cancer, a study by Timaner et al. evidenced how irradiated tumour cells generate microparticles containing high amounts of PD‐L1. When injected in recipient mice or used to treat CD8+ T cells in vitro, these microparticles induced tumour growth and T cells inhibition, respectively. These effects were partly attributed to the expression of PD‐L1 given that mitigation of the immune inhibition was observed when the PD‐1/PD‐L1 interaction was blocked (Timaner et al., 2020). Furthermore, previously irradiated tumour apoptotic bodies delivered tolerogenic signals to CD8+ T cells in vitro and in vivo, triggering the emergence of an anergic status. Analysis of their proteome showed enrichment of TGFβ‐1 on the surface resulting from the activation of the transcriptional regulator NF‐AT which upregulated its promoters (Xie et al., 2009). These new findings showed how the contribution of non‐exosomal tumour EVs to immune modulation is not trivial and needs to be addressed concurrently with exosomal research.

## 9 CONCLUSION

EVs elicit a plethora of functions in the TME and represent one of the main means of communication between cancer cells and the surrounding cells of the TME. The interaction between cancer cells and immune cells is of particular interest since the cargo message carried by EVs seems to have a central role in hijacking the immune system or even re‐purposing immune cells to promote cancer growth and dissemination. A better understanding of how different types of EVs contribute to different aspects of the anti‐cancer immune response is of paramount importance to design more effective immunotherapies for cancer patients. While several aspects of the fascinating biology of cancer derived and NK‐ and CTL‐derived EV have been unravelled, there are still several questions that need to be addressed. First, whether there is a main component (microRNAs vs. proteins, vs. DNA, vs. long non‐coding RNAs, vs. lipids or sugars) in the cargo or surface of professional killer cells’ derived EVs that affects their ability to kill cancer cells or whether the killing results from a combination of different types of molecules still needs to be established. Secondly, it is unclear how many EVs and how frequently they need to be administered in order to achieve a significant anti‐cancer effect, and how such a schedule of administration can be modified to improve the clinical outcome. Thirdly, is there a sub‐type of EVs which is better than others to achieve a therapeutic goal? Finally, how NK‐ and CTL‐derived EVs can be engineered to further improve their efficacy and how they can be directed specifically to the target cells, preventing or limiting possible side effects? While these questions need to be answered before we can translate the preclinical evidence to the clinics, it remains the fact that EVs from professional killer cells hold several possible advantages over the use of the immune cell themselves for therapeutics: in primis, EVs likely do not undergo the same receptor‐mediated immunoescape mechanisms that immune cells would undergo; moreover, they probably have an easier time to cross anatomical barriers (such as brain blood barrier and testis blood barrier) than immune cells and the preliminary in vivo evidence did not show significant incidence of ‘cytokine storm’—like symptoms in animals treated with NK— and CTL‐derived EVs. We are witnessing the dawn of a fascinating new chapter in cancer biology and therapeutics, in which immune cell‐derived EVs are the main character. The next chapter is to try to address the above questions, in order to determine whether an EV‐based immunotherapy (alone or in combination with other regimens) represents a valuable additional weapon in the war against cancer and will improve the prognosis of cancer patients.

## CONFLICTS OF INTEREST

The authors do not have any conflicts of interest to declare.
